# Actual and preferred contraceptive sources among young people: findings from the British National Survey of Sexual Attitudes and Lifestyles

**DOI:** 10.1136/bmjopen-2016-011966

**Published:** 2016-09-26

**Authors:** Rebecca S Geary, Caroline Tomes, Kyle G Jones, Anna Glasier, Wendy Macdowall, Jessica Datta, Pam Sonnenberg, Kaye Wellings, Rebecca S French, Catherine H Mercer, Anne M Johnson

**Affiliations:** 1 Research Department of Infection and Population Health, University College London, London, UK; 2 Norfolk and Norwich University Hospitals NHS Foundation Trust, Norfolk, UK; 3 Department of Social and Environmental Health Research, London School of Hygiene and Tropical Medicine, London, UK

**Keywords:** Contraceptive Service Use, Sexual Behaviour, Sexual Health, Young People, Britain

## Abstract

**Objective:**

To describe actual and preferred contraceptive sources among young people in Britain and whether discordance between these is associated with markers of sexual risk behaviour or poor sexual health.

**Design:**

Cross-sectional probability sample survey.

**Setting:**

British general population.

**Participants:**

3869 men and women aged 16–24 years interviewed for the third National Survey of Sexual Attitudes and Lifestyles (Natsal-3) between 2010 and 2012.

**Main outcome measures:**

Reported source of contraceptive method(s) and preferred source if all were available and easily accessible.

**Results:**

Of the 75% of young people (aged 16–24) who were heterosexually active (1619 women, 1233 men), >86% reported obtaining contraceptives in the past year. Most common sources were general practice (women, 63%) and retail (men, 60%): using multiple sources was common (women 40%, men 45%). Healthcare sources were preferred by 81% of women and 57% of men. Overall, 32% of women and 39% of men had not used their preferred source. This discordance was most common among men who preferred general practice (69%) and women who preferred retail (52%). Likelihood of discordance was higher among women who usually used a less effective contraceptive method or had an abortion. It was less likely among men who usually used a less effective method of contraception and men who were not in a steady relationship.

**Conclusions:**

Most young people in Britain obtained contraception in the past year but one-third had not used their preferred source. Healthcare sources were preferred. Discordance was associated with using less effective contraception and abortion among young women. Meeting young people's preference for obtaining contraception from healthcare sources could improve uptake of effective contraception to reduce unwanted pregnancies.

**Strengths and limitations of this study**

- This study uses data from a nationally representative British population-based survey to explore actual and preferred contraceptive sources among young people and whether not accessing a preferred source is associated with markers of sexual risk behaviour.
- Using national probability sample survey data, we were able to estimate the proportion of young people not accessing sources of contraception, and those using retail sources, whereas much previous research has been conducted in health service settings, omitting these groups.
- Our results may reflect patterns of contraceptive use but the data collected do not allow us to establish which method was supplied by each source.
- Information on the reasons why young people use and prefer particular sources, barriers to using their preferred source, or the contraceptive method(s) young people would prefer to obtain from each source was not collected. This may be more suited to qualitative investigation.
- After weighting, our sample was comparable with the 2011 census in terms of key demographics; however, we cannot be sure that those who participated are representative of the general population.

## Introduction

Young people are more likely to use contraception inconsistently and to have higher rates of unplanned pregnancy and abortion and lower levels of healthcare usage than older adults.^1–3^ In England, under 18 conception rates fell by 51% between 1998 and 2014 to their lowest levels since records began (1969).^4^ ^5^ However, they remain among the highest in Western Europe^6^ ^7^ at 22.9 conceptions per 1000 women aged 15–17 in England and Wales in 2014.^5^ The length of time heterosexually active young women may spend wanting to avert pregnancy is increasing due to an earlier age at first sexual intercourse, later ages at first cohabitation and parenthood, and smaller desired family sizes.^2^ Using an effective contraceptive method correctly and consistently is a reliable way to avoid unplanned pregnancy.

Contraceptive methods^i^ are available free of charge in Britain through the National Health Service (NHS). With the exception of sterilisation, they can be obtained from most general practice (GP) surgeries, community contraception clinics, some genitourinary medicine (GUM) clinics, sexual health clinics and some young people's services.^8^ Many areas have ‘C-Card’ schemes where young people can obtain free condoms and sexual health advice from participating pharmacies and health services^9^ and condoms can be bought in many retail settings. In addition to being available free of charge on the NHS, the emergency contraceptive (EC) pill, and less widely, the contraceptive pill, patch and ring can be purchased from some pharmacies and condoms from many retailers. The methods available at each source or service may vary; in particular, not all GPs provide long-acting reversible contraceptive (LARC) methods such as intrauterine devices (IUDs) and implants. National guidelines emphasise the importance of easy access to reproductive and sexual health services for young people that are youth-friendly through whole system commissioning.^9^ ^10^ They recommend the provision of the full range of contraceptives, including EC and LARC, advice on consistent use and information on benefits and side effects.^11^

Meeting young people's needs for contraceptive services may help them to avoid unplanned pregnancies. However, much research on young people's contraceptive service use in Britain has been conducted within health services, omitting non-users of services and those who use retail sources, or has been unable to examine young people's preferences. Understanding young people's preferences for contraceptive sources, how these compare to actual use, and whether not accessing a preferred source is associated with markers of poor sexual health, is valuable for informing service provision. To address this, our objectives are to present gender-specific estimates from a large national probability sample survey of the prevalence of use of different sources of contraceptives among young people, their preferred sources, and discordance between actual and preferred source, and identify sociodemographic, reproductive and sexual health and behaviour factors associated with this discordance.

## Methods

Full details of the methods used in the third National Survey of Sexual Attitudes and Lifestyles (Natsal-3) have been reported elsewhere.^12^ ^13^ Briefly, we used a multistage, clustered, stratified probability sample design. Men and women aged 16–74 years, resident in a household in Britain (England, Scotland or Wales) were interviewed between September 2010 and August 2012 (n=15 162). We oversampled individuals aged 16–34 years to allow detailed exploration of behaviours in the age group at highest risk of some sexual health outcomes such as unplanned pregnancy. Sampled addresses were randomly assigned to the core sample (where all individuals aged 16–74 were eligible) or the boost sample (where only those aged 16–34 were eligible). Participants were interviewed in their own homes through a combination of face-to-face computer-assisted personal interviews (CAPI) and computer-assisted self-interview (CASI) for the more sensitive questions. CAPI questions included those about use of, and preference for, sources of contraception. Item non-response in Natsal-3 was typically below 0.5% for questions asked in the CAPI and around 1–3% for those asked in the CASI.^14^ The denominator for this study is young men and women aged 16–24 years who reported vaginal intercourse in the past year.

We weighted the data to adjust for the unequal probabilities of selection in terms of age and the number of adults in the eligible age range at an address. After application of these selection weights, the Natsal-3 sample was broadly representative of the British population compared with 2011 Census figures, although men and London residents were slightly under-represented.^15^ ^16^ Therefore, we also applied a non-response poststratification weight to correct for differences in gender, age and region between the achieved sample and the 2011 Census. Participants provided oral informed consent to take part in interviews.

### Measures

In the CAPI, participants who reported that they or any partner had used any contraceptive method(s) together in the past year were shown a card listing different sources of contraceptive supplies and asked to indicate which source(s) they had used in that period. However, data on which contraceptive method(s) had been obtained from each source in the past year were not collected. A separate card was then shown (again only to those who reported that they or any partner had used any contraceptive method(s) together in the past year) which asked which source they would prefer, assuming all those listed were available and easy to get to in their area. Participants could report multiple sources used and one preferred source. For analysis, the sources listed were grouped into GP (doctor or nurse), community clinic (GUM/family planning/contraceptive or reproductive health clinic), youth services (eg, Brook clinic), retail (pharmacy/chemist, website, petrol station/supermarket/other shop, vending machine or mail order) and other. We considered there to be discordance between actual and preferred sources if participants did not report using their preferred source, alone or in addition to other sources, to obtain contraception in the past year.

Usual contraceptive method was derived from responses to the question ‘Which would you say is your most usual method these days?’. For analysis, reported usual method of contraception used in the past year was classified according to the most effective method reported. Methods with a typical use failure rate (including incorrect and inconsistent use) below 10% were classified as more effective (IUD, intrauterine systems (IUS), implant, injection, patch and oral contraceptives^17^). Those with a typical use failure rate of more than 10% were classified as less effective (condoms (male and female), diaphragm, pessaries, gels, EC, withdrawal and the rhythm method^17^). Grouping contraceptive methods into these categories avoided small numbers for less commonly reported methods. Data were not collected on the source from which each method was obtained.

### Statistical analyses

We used Stata (V.14) for complex survey analysis to incorporate weighting, clustering and stratification of the Natsal-3 data. The population of interest was heterosexually active young people aged 16–24 years, defined as those who reported heterosexual vaginal sex in the past year. Participants eligible for the CASI (defined as those who reported any sexual experience) were asked (in the CASI) how long ago their last occasion of vaginal sex was. Only individuals who reported that they or any partner had used any contraceptive method(s) together in the past year were routed to the questions on the source(s) they had used to obtain contraception, and where they would prefer to obtain contraceptives from. We present descriptive statistics by gender to establish which sources young people reported using, and those that they would prefer to use, to obtain contraception. We used binary logistic regression to calculate ORs to investigate how reported discordance between actual service use and preference varied by key sociodemographic and sexual and reproductive health and behaviour factors. We used multivariable logistic regression to adjust for the confounding effects of age, rural/urban location, deprivation (Index of Multiple Deprivation, area level^18^), educational attainment (defined according to school leaving age and academic qualifications obtained (individual level)), usual contraceptive method (past year), use of emergency contraception (ever), age at first sex, sexual competence at first heterosexual sex (a constructed variable to measure readiness, combining responses to questions on consensuality, autonomy of decision-making, timing and use of effective contraception^19^), number of sexual partners (past year), relationship status, frequency of sexual intercourse (past month), sexually transmitted infection (STI) diagnosis (past year), unsafe sex and, among women, pregnancy (ever) and abortion (ever). Unsafe sex was defined as not using a condom at first sex with a new (vaginal or anal sex) partner in the past year or two or more sexual partners in the past year and no condom use in that time. Those who had only oral sex with partners in the past year were classified as not having had unsafe sex.

We further restricted the denominator to those also reporting not intending to become pregnant and repeated all analyses as a sensitivity analysis. Pregnancy intention was derived from a question asking participants how they felt about having (more) children (in the CASI) with the response options: definitely like (more) and currently trying; definitely like (more) but not currently trying; might like (more) but not sure yet; definitely not like (more) and do not know. Those reporting that they were currently trying to become pregnant were classified as intending to become pregnant. All other response options were classified as not intending to become pregnant. We consider men and women separately throughout, reflecting gender differences in the reporting of contraceptive service use, as well as in the experience and reporting of sexual behaviours, and the ‘sexual scripts’ which shape behaviours.^13^ ^20^ ^21^

### Role of the funding source

The funders of the study had no role in the study design, data collection, data analysis, data interpretation or writing of the report. The corresponding author had full access to all the data in the study and had final responsibility for the decision to submit for publication.

## Results

Three-quarters of young people aged 16–24 years reported having had heterosexual vaginal sex in the past year, corresponding to a sample of 1614 young women and 1231 young men for these analyses. Of these heterosexually active participants, 93% of women and 86% of men reported having used at least one source to obtain contraceptives in the past year (table 1). The most commonly reported source of contraception among young women was GP (63%) and among young men, retail (60%). Community clinics were the second most commonly reported source (young women, 35%, young men, 31%). Almost half of the young men (45%) and 40% of women reported having used more than one source for contraceptive supplies in the past year. Those who reported using more than one source were also more likely to report having used more than one contraceptive method (data not shown). Seven per cent of young women and 14% of young men had not obtained supplies of contraception in the past year (table 1). Of these, approximately one-quarter of women (25%) and men (23%) reported usually using LARC and therefore may have not required supplies in the past year. A further half of those reporting not having obtained contraceptive supplies in the past year reported usually using oral contraceptives (54% of men) or condoms (56% of women) and not obtaining these methods may be explained by a partner taking responsibility for this.

**Table 1:** Sources used and preferred for contraceptive supplies by gender

	Young women	Young men
	Percentage (95% CI)	Percentage (95% CI)
Denominators (unweighted, weighted)	1614, 896	1231, 905
Source(s) used*		
General practice	63.0 (60.1 to 65.8)	15.9 (13.6 to 18.6)
Community clinic	35.3 (32.6 to 38.2)	30.6 (27.7 to 33.6)
Retail	26.8 (24.3 to 29.5)	59.6 (56.3 to 62.8)
Youth services	9.7 (8.1 to 11.6)	22.2 (19.6 to 25.1)
Other	3.0 (2.2 to 4.1)	5.5 (4.3 to 7.1)
None	7.2 (5.8 to 8.9)	13.6 (11.5 to 16.0)
Number of sources used		
0	7.2 (5.8 to 8.9)	13.6 (11.5 to 16.1)
1	52.6 (49.7 to 55.5)	41.0 (37.9 to 44.0)
2	29.1 (26.6 to 31.8)	25.4 (22.6 to 28.4)
3+	11.1 (9.4 to 13.0)	20.1 (17.4 to 23.1)
Preferred source		
General practice	46.9 (44.0 to 49.8)	20.5 (18.0 to 23.2)
Community clinic	29.8 (27.3 to 32.5)	29.3 (26.4 to 32.3)
Youth services	4.6 (16.4 to 21.2)	7.3 (39.7 to 46.2)
Retail	18.7 (3.5 to 6.0)	42.9 (5.7 to 9.3)
Discordance between actual and preferred source		
No	67.7 (65.0 to 70.4)	60.8 (57.4 to 64.1)
Yes	32.3 (29.7 to 35.1)	39.2 (36.0 to 42.6)

Denominator: young people (aged 16–24 years) who reported having had vaginal intercourse in the past year.

*Not mutually exclusive; young people could report use of more than one source.

**Figure 1:**
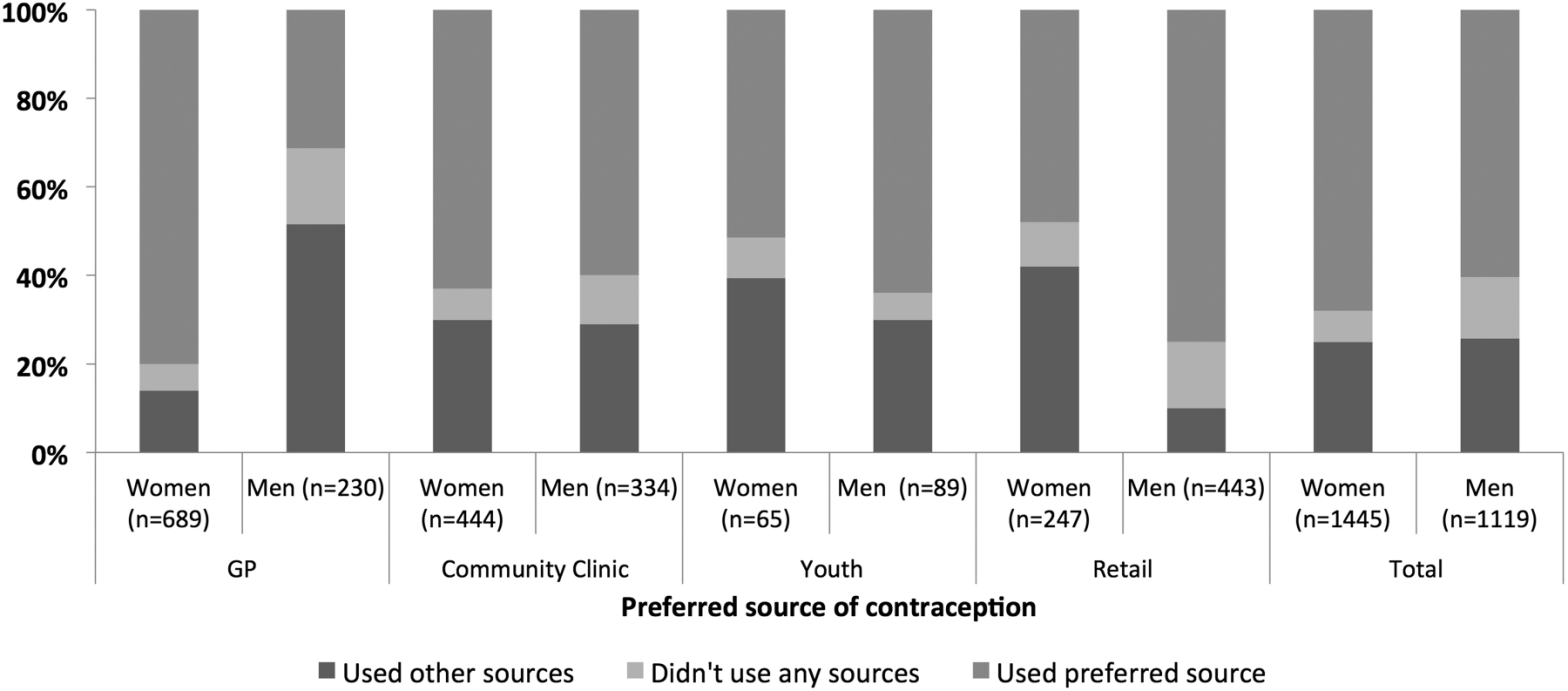
Discordance between actual and preferred source of contraception among young people by preferred source and gender. GP, general practice.

For both women and men, the most commonly used source was also the most preferred; 47% of women preferred GP while 43% of men preferred retail sources. There was substantial discordance between actual and preferred source of contraception; 32% of young women and 39% of young men reported not having used their preferred source in the past year (table 1). The lowest level of discordance between actual and preferred source was among women who preferred GP and men who preferred retail (19% and 25%, respectively, figure 1).

Discordance between actual and preferred source was highest among young men who preferred GP and young women who preferred youth services or retail (70%, 53% and 51%, respectively, figure 1). Although 16% of young men preferred GP (table 1), just one-third of these young men reported having used this source (figure 1). The majority of young men who preferred GP used retail sources (data not shown), which may reflect limited availability of condoms in GP. A sizeable percentage of young women preferred retail (27%, table 1) but less than half of these had used this source in the past year (figure 1). The majority of these young women used GP, alone or in combination with other sources (data not shown). More than 15% of young men who preferred GP or retail reported not having obtained contraceptives in the past year, but their partner(s) may have done so. Youth services were the least preferred source (<10% of men and <5% of women, table 1). However, the majority of young men (63%) and almost half of the young women (47%) who preferred youth services reported having used this source (figure 1).

Among young women, discordance between actual and preferred source of contraception was more likely among those who reported usually using a less effective contraceptive method (adjusted OR (AOR) 1.94, 95% CI 1.46 to 2.59) and those who had ever had an abortion (AOR 1.82, 95% CI 1.16 to 2.86, table 2). Among young men, discordance was less likely among those not in a steady relationship (AOR 0.65, 95% CI 0.43 to 0.96) and those who reported usually using a less effective contraceptive method (AOR 0.72, 95% CI 0.53 to 0.99). Age group, area-level deprivation, urban/rural location, educational attainment, age and sexual competence at first sex, use of emergency contraception (ever), pregnancy (ever) and STI diagnoses in the past year were not associated with discordance.

**Table 2:** Associations between discordance of sources used and preferred for contraceptive supplies and sexual behaviours, by gender

Discordance between actual and preferred source of contraception	Young women				Young men			
		32.3%	(29.7, 35.1)	p Value	Denominators	39.2%	(36.0, 42.6)	p Value
	Denominators (unweighted, weighted)	OR	AOR (95% CI)			OR	AOR (95% CI)	
Age group				0.0781				0.8573
16–19	479, 245	1	1		368, 243	1	1	
20–24	832, 483	1.20	1.64 (0.95 to 2.85)		627, 497	1.08	1.06 (0.55 to 2.05)	
Urban or rural resident				0.8796				0.7264
Rural or town area (<10 000)	230, 124	1	1		219, 147	1	1	
Urban area (>10 000)	1081, 604	1.10	0.97 (0.68 to 1.39)		776, 593	0.98	1.06 (0.75 to 1.51)	
Quintile of index of multiple deprivation				0.701				0.6307
1 (least deprived)	235, 134	1	1		187, 136	1	1	
2	243, 137	0.99	0.91 (0.58 to 1.42)		199, 148	0.96	1.11 (0.72 to 1.72)	
3	242, 143	1.07	1.01 (0.65 to 1.58)		190, 140	0.82	0.93 (0.58 to 1.49)	
4	277, 154	0.98	0.85 (0.55 to 1.32)		208, 171	0.78	0.86 (0.53 to 1.39)	
5 (most deprived)	314, 159	1.19	1.12 (0.73 to 1.72)		211, 145	1.13	1.21 (0.76 to 1.92)	
Academic qualifications*				0.4503				0.0912
Studying for/attained further academic qualifications	828, 483	1	1		613, 472	1	1	
No qualifications or academic qualifications typically gained age 16†	483, 245	1.22	1.13 (0.82 to 1.55)		382, 268	1.25	1.31 (0.96 to 1.78)	
Usual method of contraception in the past year				0				0.0452
Effective	933, 513	1	1		454, 329	1	1	
Less effective/no method	378, 215	2.10‡	1.94 (1.46 to 2.59)		541, 410	0.67‡	0.72 (0.53 to 0.99)	
Ever used emergency contraception				0.3339				0.3219
No	807, 451	1	1		631, 473	1	1	
Yes	504, 277	0.90	0.87 (0.65 to 1.16)		364, 267	0.77	0.85 (0.63 to 1.17)	
Unsafe sex in past year∼				0.4515				0.1885
No	899, 512	1	1		704, 529	1	1	
Yes	412, 216	0.95	0.87 (0.62 to 1.24)		291, 211	1.01	1.26 (0.89 to 1.78)	
First heterosexual sex before age 16				0.8355				0.1183
First sex after age 16	514, 260	1	1		386, 270	1	1	
First sex before age 16	797, 468	1.04	0.97 (0.72 to 1.31)		609, 469	1.28	1.31 (0.93 to 1.83)	
Sexual competence at first heterosexual sex				0.8107				0.5895
Not competent	671, 362	1	1		404, 303	1	1	
Competent	640, 366	0.97	0.97 (0.72 to 1.29)		591, 436	1.04	1.09 (0.80 to 1.49)	
Number of sexual partners in the past year				0.3386				0.1018
1	802, 452	1	1		535, 401	1	1	
≥2	509, 276	1.07	1.18 (0.84 to 1.66)		460, 338	0.63‡	0.75 (0.53 to 1.06)	
Relationship status				0.7836				0.0309
In a steady relationship	910, 509	1	1		562, 424	1	1	
Not in a steady relationship	401, 219	1.34‡	1.05 (0.74 to 1.48)		433, 315	0.69‡	0.65 (0.43 to 0.96)	
Number of occasions of heterosexual sex in the past 4 weeks				0.1098				0.0633
0–2	466, 262	1	1		431, 320	1	1	
≥3	845, 466	0.67‡	0.78 (0.57 to 1.06)		564, 419	0.97	0.71 (0.49 to 1.02)	
STI diagnosis in the past year				0.2192				0.1407
No	1247, 693	1	1		964, 717	1	1	
Yes	64, 35	0.70	0.71 (0.41 to 1.23)		31, 22	0.43‡	0.47 (0.18 to 1.28)	
Ever been pregnant				0.5222				
No	824, 491	1	1					
Yes	487, 237	1.15	0.89 (0.61 to 1.28)					
Ever had an abortion				0.0092				
No	1153, 649	1	1					
Yes	158, 79	1.47‡	1.82 (1.16 to 2.86)					

Denominator: young people (aged 16–24 years) who reported having had vaginal intercourse in the past year.

Multivariable logistic regression adjusted for the confounding effects of age, rural/urban location, deprivation, education attainment, contraceptive method used in the past year, ever use of emergency contraception, unsafe sex in the past year, age at first sex, sexual competence at first heterosexual sex, number of sexual partners in the past year, relationship status, frequency of sexual intercourse, STI symptoms and, among women, pregnancy (ever) and abortion (ever). *Participants aged ≥17 years.

†English General Certificate of Secondary Education or equivalent.

‡p<0.05 in univariate analyses.

∼, Unsafe sex defined as not using a condom at first sex with a new (vaginal or anal sex) partner in the past year or two or more sexual partners in the past year and no condom use in that time; AOR, adjusted OR; STI, sexually transmitted infection.

### Sensitivity analysis

Of the 1614 women and 1231 men and who reported vaginal sex in the past year, 95% of these men and 94% of these women reported not intending to get pregnant at the time of interview. Of those who reported that they were trying to get pregnant, 83% of women and 72% of men also reported having obtained contraception from at least one source. Excluding those intending to get pregnant did not change the results with the exception that the association between discordance and abortion among women was not statistically significant in multivariable analyses and the association between discordance and number of occasions of heterosexual sex (past month) was statistically significant among men (see online supplementary appendix table 1).

10.1136/gutjnl-2015-311146.supp1 Supplementary appendix

## Discussion

Our study shows that most heterosexually active young people in Britain used at least one source to obtain contraceptives, but one-third had not used their preferred source. Young people preferred healthcare sources, particularly GP. We found that discordance between actual and preferred contraceptive source was associated with the contraceptive method usually used and, for women, with having had an abortion.

### Strengths and limitations

Much research on contraceptive service use has been conducted within health service settings, omitting non-users of services and those who use retail sources. By using national probability sample survey data, we were able to estimate the proportion of young people not accessing sources of contraception, and those using retail sources. The response rate in Natsal-3 is in line with other major social surveys completed in Britain during this period.^22^ ^23^ However, we acknowledge that non-response could be a source of bias in these data. We aimed to minimise this by weighting the sample to be broadly representative of the British general population in terms of gender, age and region, based on Census 2011. Our results may reflect patterns of contraceptive use but the data collected do not allow us to establish which method was supplied by each source, or why a particular source was used or preferred. No information was given to participants on how to report sources for methods such as the oral contraceptives, which may have been prescribed by a GP but the prescription may have been filled by a pharmacy. How this was reported is therefore likely to depend on the individual participant. These data do not allow us to capture whether young men and women obtained contraceptives together. In addition, the observed gender differences may in part reflect some men not reporting methods used by their female partner, and vice versa for women, although the question did ask participants to report methods that ‘they or any partner had used together’. Some men may not have known about a partner's contraceptive method use. Furthermore, those who reported not using contraception in the past year were not asked about their preferred source. Only one online source (NHS/Department of Health website) was included in the response options for preferred source. The lead time required to design and implement nationally representative probability sample surveys means that rapidly evolving areas may not always be fully captured. The data do not allow us to investigate why young people use and prefer given sources, the barriers to using their preferred source, or which particular contraceptive method(s) they would prefer to obtain from which source. Further research on these topics, including qualitative studies, could facilitate the understanding of how contraceptive policy and provision could meet these preferences, where appropriate and feasible. We define unsafe sex as not using a condom at first (vaginal or anal) sex with a new partner in the past year or having two or more sexual partners in the past year and no condom use in that time. Those reporting only oral sex with partners in the past year were classified as not having had unsafe sex. Other definitions of unsafe sex could be used. Self-reported condom use may be affected by social desirability bias leading to underestimates of unsafe sex. Many STIs are asymptomatic, which could bias any association between STI diagnosis in the past year and discordance between actual and preferred source of contraception towards the null. STI diagnosis was not associated with discordance between actual and preferred source of contraception regardless of the time frame considered (STI diagnosis in the past year, past 5 years or ever). The data presented are representative of individuals aged 16–24 living in private residential households only; younger adolescents, the homeless and those living in institutions may have different profiles of actual and preferred sources of contraception.

The inclusion in Natsal-3 of questions on fertility intentions allowed us to build on analyses of Natsal-2^24^ by comparing results among all heterosexually active young people with those not trying to get pregnant. The majority of young people who reported that they were trying to get pregnant had obtained contraceptives in the past year and hence were included in our main analysis. Participants were asked how they felt about having (more) children, at the time of the interview, so reporting trying to get pregnant (at interview) is not incompatible with having obtained contraception in the past year. Our sensitivity analysis shows similar actual and preferred sources of contraception, levels of discordance and associations between discordance and the contraceptive method usually used among all heterosexually active young people and those not trying to get pregnant.

Our findings support those of earlier studies which found that the main source of contraception was GP for young women and the retail sector for young men.^24^ ^25^ An evaluation of England's Teenage Pregnancy Strategy found that young men's use of GP for contraception increased significantly between 2000 and 2004, while their use of retail sources declined.^25^ Our results suggest a continued increase in young women's and men's use of GP to obtain contraceptives, although the data do not allow us to tell which methods they obtained there. Among the healthcare sources, youth services were the least commonly used. However, some youth services may not be available to those over 18 and may target those under 16, who are not included in Natsal-3. Youth services may reach more at-risk groups.^26^ ^27^

Using multiple sources may be driven by which source and/or method works best for an individual generally, and at a given relationship stage. It may reflect individuals trying a number of sources and methods to establish which they prefer and different source(s) may be used to obtain different methods. Use of multiple sources to obtain contraception appears to have increased among young people between 1999–2001 and 2010–2012. Approximately 25% of women and 14% of men (16–24 years) reported having used multiple sources for contraceptive supplies or advice in 1999–2001,^24^ compared with 46% of young men and 40% of young women using multiple sources for supplies alone in 2010–2012. This may reflect wider method choices offered by different services and by public health efforts to address myths about which methods young people can use.^11^ ^28^ In addition, LARC methods are not consistently available in GP, so people approaching this source for these methods may be referred elsewhere. This may affect the number of sources used, which source is preferred and which method is used.

The provision of online sexual health services, including for contraception, is expanding in Britain and other countries.^29–31^ Our findings suggest that few young people use the internet to obtain contraception and that the majority would prefer to obtain contraception from a healthcare source. This is in line with findings of low reported use of the internet for STI testing in Britain.^32^ However, as provision and knowledge of online sources increase, their use may become more widespread. Levels of health service usage among young people are often lower and more variable than among older adults, with young men often reporting particularly low levels of usage. Young men have described barriers to seeking healthcare including masculinity norms, the perception that some services emphasise women's health and fear of stigma and disrespect.^33–35^ Our findings, however, appear to indicate that, despite the barriers young men face in accessing sexual health services, they would like to access them for condoms and/or information around sexual health. This suggests opportunities exist to engage young men in healthcare services if their preferences can be better understood. This could also inform provision of free condom schemes and sex and relationships education. Packaging contraception services and STI/HIV prevention as part of a more holistic service that can also address other sexual issues including non-volitional sex, relationships and sexual function/satisfaction may help to get young people, including young men, to engage with health services.

Our finding that discordance between actual and preferred source of contraception was associated with young people's usual method of contraception, and experience of abortion, has implications for service commissioners and practitioners. The cross-sectional nature of our study means that we cannot assume causality in these associations. However, as commissioning structures in Britain become increasingly complex, it is important for commissioners to ensure that a wide range of services provide the full choice of contraceptive methods, including LARC, to enable young people to access their preferred source and effective methods of contraception. Meeting young people's preferences for healthcare sources, particularly GP, may provide opportunities for healthcare professionals to provide information on, and supplies of, more effective contraceptive methods, as well as information on condom use and STI prevention for sexual and reproductive health gain. Previous research has found that young people want more information about psychosexual matters, STIs and, for women, contraception, when they first feel ready for sexual experience, and that they would prefer to receive this from health professionals.^36^

The reasons why young people use and prefer particular sources of contraception, and the barriers to young people using their preferred source, need exploration. An evaluation of one-stop-shop sexual health services in England, including contraceptive services, found that barriers included cost (retail), lack of method choice, opening times, appointment systems, confidentiality concerns, location and lack of knowledge.^27^ One-fifth of young women preferred retail as a source of contraception, but less than half of these women had used this source. In addition to being available for free on the NHS, EC and the contraceptive pill, patch and ring are available at some pharmacies in Britain. However, these methods may not be widely available and geographical coverage may vary. Furthermore, more effective methods such as LARC are not available from these sources. Nonetheless, understanding why some young women prefer retail sources could inform changes to make the services which can provide the full range of methods more appealing. Reasons why young men use and prefer GP also warrant investigation of whether this is driven by: a need for free condoms, seeking advice or other services and obtaining condoms simultaneously, and/or attending in support of their partner(s) obtaining contraception. Discordance between actual and preferred source may represent one or a combination of factors such as availability, accessibility or acceptability of sources and methods, and may differ by source. Future research could address these issues and establish whether contraceptive source is linked to method satisfaction, as the latter is associated with lower levels of contraceptive discontinuation.^37^ Further work will elucidate whether discordance between actual and preferred source varies by age.

The majority of heterosexually active young people do access contraception. However, even though a range of sources provide contraception and are used by young people in Britain, one-third had not used their preferred source and this was associated with use of a less effective method of contraception among young women. This research supports the recommendation that, where possible, commissioners should ensure that a wide range of services provide the full choice of contraceptive methods, including LARC, to enable young people in their area to access their preferred source to obtain contraception.

## Footnotes

i The methods available are: the intrauterine device (IUD), the intrauterine system (IUS), oral contraceptive pills (the combined pill and the progestogen-only pill), condoms (male and female), implants, injections, the contraceptive patch, the vaginal ring, diaphragms/caps, emergency contraception (pill and IUD) and male and female sterilisation (NHS Choices. Your contraception guide 2015 [11/05/2015]. Available from: http://www.nhs.uk/Conditions/contraception-guide/Pages/contraception.aspx)
