# Supplementary material for: Actual and preferred contraceptive sources among young people: findings from the British National Survey of Sexual Attitudes and Lifestyles
Source: BMJ Open. 2016 Sep 26;6(9):e011966. doi: 10.1136/bmjopen-2016-011966 (PMC5051385; doi:10.1136/bmjopen-2016-011966)
Supplement: Supplementary appendix [file bmjopen-2016-011966supp_appendix.pdf]

	Young women					Young men				
**Discordance between actual and preferred source of contraception**		32.2	[29.5,35.0]				38.9	[35.6,42.4]		
	**Denominators (unweighted, weighted)**	**OR**	**AOR**	**95% C.I.**	**P-value**	**Denominators**	**OR**	**AOR**	**95% C.I.**	**P-value**
**Age group**					0.1163					0.7182
16-19	457, 234	1	1	-		359, 238	1	1	-	
20-24	764, 446	1.24	1.57	(0.89-2.76)		597, 477	1.1	1.13	(0.58-2.21)	
**Urban or rural resident**					0.8676					0.8019
Rural or Town area (<10,000)	221, 120	1	1	-		215, 144	1	1	-	
Urban area (>10,000)	1000, 560	1.11	1.03	(0.72-1.49)		741, 570	0.95	1.05	(0.73-1.49)	
**Quintile of Index of Multiple Deprivation**					0.7927					0.7127
Least deprived	225, 128	1	1	-		183, 133	1	1	-	
2	231, 130	0.92	0.85	(0.54-1.34)		194, 145	0.96	1.11	(0.71-1.73)	
3	230, 136	1.02	0.99	(0.64-1.54)		180, 134	0.84	0.98	(0.60-1.58)	
4	252, 140	0.94	0.8	(0.51-1.26)		198, 163	0.79	0.88	(0.54-1.44)	
Most deprived	283, 144	1.07	0.98	(0.63-1.53)		201, 139	1.1	1.23	(0.77-1.98)	
**Academic qualifications^**					0.4156					0.0769
Studying for/attained further academic qualifications	805, 470	1	1	-		597, 462	1	1	-	
No qualifications or academic qualifications typically gained age 16 †	416, 210	1.24	1.14	(0.83-1.58)		359, 253	1.26	1.33	(0.97-1.82)	
**Usual method of contraception in the past year**					0					0.06
Effective	888, 489	1	1	-		434, 317	1	1	-	
Less effective/no method	333, 190	2.15*	2.01	(1.47-2.75)		522, 398	0.66*	0.73	(0.53-1.01)	
**Ever used emergency contraception**					0.2476					0.4182
No	749, 418	1	1	-		608, 459	1	1	-	
Yes	472, 262	0.88	0.84	(0.62-1.13)		348, 255	0.78	0.88	(0.64-1.21)	
**Unsafe sex in last year**					0.9707					0.1004
No	837, 476	1	1	-		679, 512	1	1	-	
Yes	384, 204	1	0.99	(0.69-1.42)		277, 202	1.05	1.35	(0.94-1.93)	
**First heterosexual sex before age 16**					0.9012					0.0765
First sex after age 16	471, 239	1	1	-		364, 256	1	1	-	
First sex before age 16	750, 441	1.05	0.98	(0.71-1.34)		592, 459	1.35*	1.37	(0.97-1.93)	
**Sexual competence at first heterosexual sex**					0.7309					0.4669

Not competent	617, 334	1	1	-		386, 293	1	1	-	
Competent	604, 345	0.94	0.95	(0.70-1.28)		570, 421	1.08	1.13	(0.82-1.55)	
**Number of sexual partners in the past year**					0.416					0.0922
1	737, 415	1	1	-		508, 383	1	1	-	
2 or more	484, 265	1.06	1.16	(0.81-1.64)		448, 331	0.63*	0.73	(0.51-1.05)	
**Relationship Status**					0.97					0.0208
In a steady relationship	831, 467	1	1	-		527, 402	1	1	-	
Not in a steady relationship	390, 213	1.26	0.99	(0.70-1.41)		429, 313	0.71*	0.62	(0.41-0.93)	
**Number of occasions of heterosexual sex in the past 4 weeks**					0.2256					0.0218
0-2	443, 248	1	1	-		422, 315	1	1	-	
3 or more	778, 431	0.71*	0.82	(0.60-1.13)		534, 400	0.92	0.64	(0.44-0.94)	
**Any STI diagnosis in the past year**					0.2127					0.1006
No	1165, 647	1	1	-		926, 693	1	1	-	
Yes	56, 33	0.67	0.7	(0.40-1.23)		30, 22	0.36*	0.41	(0.14-1.19)	
**Ever been pregnant**					0.7872					
No	806, 479	1	1	-						
Yes	415, 201	1.16	0.95	(0.65-1.39)						
**Ever had an abortion**					0.0648					
No	1080, 608	1	1	-						
Yes	141, 72	1.35	1.58	(0.97-2.56)						

Appendix Table A1: Associations between discordance of sources used and preferred for contraceptive supplies and sexual behaviours, by gender
Denominator: young people (aged 16-24 years) who reported having had vaginal intercourse in the past year and not trying to get pregnant at interview. * P<0.05 in univariate analyses. ^Participants aged ≥17 years. †English General Certificate of Secondary Education or equivalent. Multivariable logistic regression adjusted for the confounding effects of age, rural/urban location, deprivation, education attainment, contraceptive method used in the past year, ever use of emergency contraception, unsafe sex in the past year, age at first sex, sexual competence at first heterosexual sex, number of sexual partner in the past year, relationship status, frequency of sexual intercourse, STI symptoms and, among women, pregnancy (ever) and abortion (ever). AOR=Adjusted odds ratio
